# P58 An unusual case of a swollen knee

**DOI:** 10.1093/rap/rkae117.089

**Published:** 2024-11-05

**Authors:** Madeleine Mackay, Nandita Pai, Samundeeswari Deepak, Kishore Warrier

**Affiliations:** Queens Medical Centre, Nottingham, United Kingdom; Queens Medical Centre, Nottingham, United Kingdom; Queens Medical Centre, Nottingham, United Kingdom; Queens Medical Centre, Nottingham, United Kingdom

## Abstract

**Introduction:**

This is a rare presentation of a rapidly progressive monoarthritis in a child with a complex medical background. She has short stature with growth hormone deficiency, on growth hormone replacement; microcephaly, developmental delay, anaemia, multiple allergies and previous febrile seizures. Parents are consanguineous. Genetic testing observed several regions of homozygosity (consistent with consanguinity), but Trio exome sequence analysis did not identify a genetic cause for her problems.

It outlines the importance of careful investigation, revisiting diagnosis when there is not the expected response to treatment, and how medical background may influence the differential diagnosis to consider.

**Case description:**

The patient presented at age 7 years to her local A&E with a 5-day history of left knee swelling, no pain and weight bearing with a normal gait. She was systemically well. She had a history of viral URTI one week prior. She was diagnosed with likely reactive arthritis and was followed up in orthopaedic clinic 3 weeks later. Her swelling persisted and she was referred to Rheumatology.

On review in the Paediatric Rheumatology Clinic 3 months later, she had a large effusion in the left knee, the knee was warm but had good range of movement with minimal restriction at the end of extension and no pain. There was no other joint involvement, or systemic symptoms. An urgent MRI scan and joint aspiration were arranged.

Investigations showed low Hb (108 g/L), high plt (537 × 10^9^/L), high WCC (15.2 × 10^9^/L) but normal inflammatory markers (CRP 1.2 mg/L, ESR 14 mm/hr). Her ANA was weakly positive (400). RF, Lyme serology, and QuantiFERON negative. Synovial fluid was negative for microscopy, culture, acid fast bacilli and TB culture.

MRI of her left knee showed a very large joint effusion with significant thickened and enhancing synovium; erosive osseous lesions at the lateral femoral condyle and medial tibial plateau. Appearances were unusual. Differentials included an atypical infection such as TB as well as juvenile idiopathic arthritis (JIA).

An urgent referral was sent to orthopaedics for synovial biopsy to rule out atypical infection. This showed a hyperplastic thickened synovium suggestive of chronic inflammation with calcification, no evidence of infection or PVNS.

She was treated as presumed JIA. She had minimal response to an intra-articular steroid injection to the right knee under and was started on methotrexate.

**Discussion:**

The patient was reviewed in a clinic and was noted to have a worsening valgus deformity of the left knee with instability of lateral flexion in clinic. X-ray was suggestive of periosteal reaction in the lateral aspect of distal femur with cortical disruption and prominent heterotopic ossification within the soft tissue beyond expected bony borders – progressive in appearance compared to previous images. We sought urgent orthopaedic advice and the patient was put in a hinged brace to stabilise the knee.

Urgent MRI scan of the knee showed acute on chronic joint disease with synovitis, destruction of the lateral femoral condyle, periosteal reaction, intra-articular calcification. The appearance was felt to favour a traumatic injury or osteochondral fracture rather than an inflammatory arthropathy. Her case was discussed with the Royal Orthopaedic Hospital in Birmingham, and the images were not felt to be suggestive of malignancy. As this was not felt to be JIA methotrexate was stopped.

A variety of differentials were considered: an infection that evaded standard microbiological tests; initial trauma leading to reactionary changes; weakened bone fracture related to growth hormone deficiency and replacement therapy and epilepsy medication; or an extremely aggressive & destructive treatment resistant inflammatory arthropathy

Given the Charcot knee, and global developmental delay concerns were raised whether the patient could have an inherited sensory neuropathy. She was referred to Genetics, however they were not able to confirm a monogenetic form of congenital insensitivity to pain, or other plausible genetic cause to explain her problems.

There was a detailed multicentre radiology review and when correlating clinical history, progression, blood reports and joint aspiration results, radiological appearances favour a diagnosis of osteonecrosis of the lateral femoral condyle with secondary osteochondral fracture, synovitis and reactive new bone formation.

**Key learning points:**

• This case has outlined the importance of thorough investigation of a monoarthritis, particularly when rapidly progressive and not responding to treatment. It also highlights the importance of considering the whole patient, their medical background, medications and how this could influence their presentation.

• In this case our patient has a complex background with consanguineous parents, which makes the possibility of an underlying genetic diagnosis higher. Although no causative gene has been found, this does not rule out the possibility that the patient’s problems are genetic in origin.

• We should consider the possibility that medications such as antiepileptics or growth hormone may have contributed to poor bone health and an increased risk of osteochondral fracture. In this case when images were reviewed in retropect the osteochondral fracture was visible earlier than was reported.

**The key learning points are:**

• Monoarthritis requires careful investigation and consideration of potential differential diagnosis such as atypical arthritis, PVNS, and malignancy.

• If patients do not respond to standard treatment of JIA the diagnosis should be reviewed

• Osteochondral fracture should be considered as a differential, even without a history of trauma in children with complex background who may be predisposed to osteopenia due to medications.

